# Determination of Trace Elements in Maize Flour Consumed in Turkey by ICP-OES and Assessment of Non-Carcinogenic Health Risk

**DOI:** 10.1007/s12011-026-05174-7

**Published:** 2026-06-15

**Authors:** Erkan Kırıs, Nilay Akcay

**Affiliations:** Department of Physics, Faculty of Arts and Sciences, Recep Tayyip Erdogan University, Rize, Turkey

**Keywords:** Maize flour, Trace elements, ICP-OES, Statistical analysis, Non-carcinogenic risk

## Abstract

Maize flour is widely consumed in Turkey and may contain trace elements originating from agricultural practices and environmental contamination during cultivation, processing, and storage. This study aimed to determine the concentrations of Fe, Zn, Cu, Ni, Mn, Cd, and Cr in 20 commercially available maize flour samples using Inductively Coupled Plasma–Optical Emission Spectrometry (ICP-OES). In addition, potential non-carcinogenic health risks associated with maize flour consumption were evaluated. The accuracy and reliability of the analytical procedure were verified using certified reference material analysis and quality control procedures. The mean concentrations of Fe, Zn, Cu, Ni, and Mn in the analyzed samples were found to be 18.5, 13.1, 1.7, 0.2, and 3.9 mg/kg, respectively, while Cd and Cr concentrations were below the detection limits. The obtained concentrations were evaluated according to international regulatory limits and previously reported literature data. Non-carcinogenic health risk assessment was conducted using hazard quotient (HQ) and hazard index (HI) values, and the results indicated that the detected trace element concentrations are unlikely to pose significant non-carcinogenic health risks to consumers. In addition, descriptive statistical analysis, Spearman’s rank correlation analysis, and Principal Component Analysis (PCA) were performed to evaluate the distribution characteristics of the analyzed elements, investigate relationships among trace elements, and identify possible common sources. These findings may provide baseline data for future studies on trace element levels and dietary exposure assessment of maize flour consumed in Turkey.

**Graphical Abstract:**

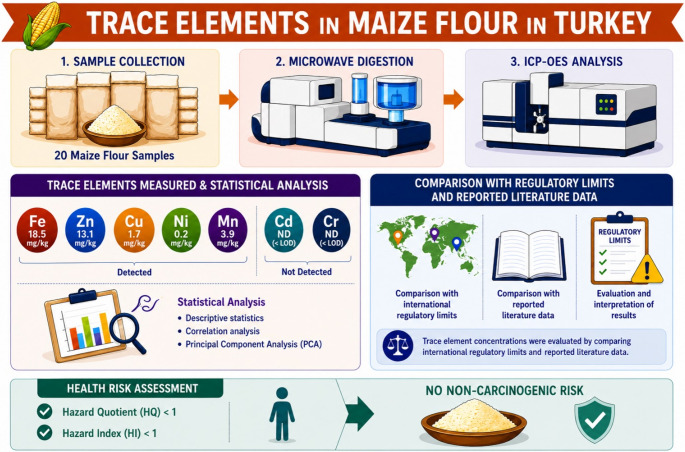

## Introduction

Maize (*Zea mays* L.), commonly referred to as corn, is one of the most widely consumed cereal crops worldwide and is extensively used in the production of foods for human consumption. According to the Food and Agriculture Organization of the United Nations, global maize consumption exceeds 150 million tons annually [1]. In addition to its economic importance, maize is recognized for its nutritional value due to its content of B-group vitamins and other essential nutrients involved in carbohydrate, lipid, and protein metabolism [2]. Maize is also among the major cereal crops produced in Turkey, with an annual production of approximately 9 million tons, corresponding to 11.6% of the country’s total cereal and other crop production according to 2023 TÜİK data [3]. Due to its widespread consumption and nutritional contribution, maize is processed into various food products; among them, maize flour is widely used in the production of bread, breakfast foods, and related products. It is one of the most commonly consumed cereal flours in Turkey, particularly in the Black Sea region, where it is traditionally produced in local mills and consumed as part of the daily diet [4–6].

Trace elements such as Fe, Zn, Cu, and Mn are essential micronutrients for human health and play critical roles in plant metabolic and biosynthetic processes as enzymatic cofactors. Zn, Mn, and Fe participate in numerous enzymatic reactions, whereas Cu is associated with amino acid complexes and antioxidant defense systems [7, 8]. Although these elements are required for normal biological functions, excessive concentrations may lead to toxic effects [9]. Ni is also considered an essential element for plant growth and microbial activity; however, elevated concentrations may adversely affect photosynthesis in plants and contribute to chronic health disorders in humans [10]. In addition to essential trace elements, potentially toxic elements such as Cd and Cr are of concern due to their persistence in the environment, adverse effects on plant growth, and risks to human health through the food chain [11–13]. Agricultural crops may be exposed to trace elements through direct uptake from soil, water, and air, as well as through indirect contamination pathways associated with atmospheric deposition and contaminated environmental sources [14, 15]. Consequently, these elements may accumulate in crops intended for human consumption, and their presence at elevated concentrations in agricultural products and processed foods may pose significant risks to food safety and public health [16].

The elemental composition of maize kernels may be influenced by kernel variety, environmental conditions, soil properties, and climatic factors. In addition, contamination during cultivation, processing, and storage may affect the trace element content of maize flour. Variations in production technologies and geographical conditions may also contribute to differences in the elemental composition of maize-derived products [1]. Therefore, monitoring trace element concentrations in maize flour and evaluating their potential health risks are essential for ensuring food safety. Several studies have investigated the occurrence of trace elements in maize flour. Maina et al. determined the concentrations of Mn, Fe, Cu, and Zn in maize flour samples collected from supermarkets in Nairobi, Kenya, and estimated the daily intake of these elements [17]. Filho et al. reported the concentrations of essential elements (Ca, Cu, Fe, Mn, Mg, and Zn) and non-essential metals (Cd and Cr) in yellow and white maize flour samples from Paraná State, Brazil [1]. Similarly, Al-Trbany et al. investigated essential trace elements (Fe, Mn, Zn, Cu, Co, Ni, and Sn) together with toxic metals (Pb, Cd, and Cr) in maize flour samples collected from different regions of Egypt and compared the measured concentrations with international permissible limits [2].

Considering the widespread consumption of maize flour in Turkey and its potential exposure to trace elements originating from environmental and processing-related sources, evaluating the concentrations of these elements is of considerable importance. Therefore, systematic monitoring is necessary not only to ensure food safety and protect public health but also to support regulatory decision-making related to maize production and processing. However, limited information is available regarding trace element contamination and associated health risks in maize flour products consumed in Turkey.

In this study, the concentrations of Fe, Zn, Cu, Mn, and Ni in commercially available maize flour samples were determined using inductively coupled plasma optical emission spectrometry (ICP-OES), while Cd and Cr were found to be below the detection limits of the analytical method. In addition, potential non-carcinogenic health risks associated with dietary exposure were evaluated using hazard quotient (HQ) and hazard index (HI) calculations. Descriptive statistical analyses were performed to evaluate the distribution characteristics and variability of the analyzed elements in maize flour samples, while Spearman’s rank correlation analysis and Principal Component Analysis (PCA) were used to investigate relationships among trace elements and identify possible common sources. Therefore, the present study provides comprehensive information on trace element concentrations, relationships among trace elements, possible common sources, and potential non-carcinogenic health risks associated with maize flour consumption in Turkey.

## Materials and Methods

### Sample Collection and Preparation

A total of 20 commercially available yellow maize flour samples from different domestic brands, all produced in Turkey, were purchased via online marketplaces in March 2021. According to the information provided on the product labels, the shelf life of the collected maize flour products varied between 6 and 24 months. In the laboratory, the samples were dried to constant weight and sieved to obtain a uniform particle size. Each sample was subsequently homogenized and stored under refrigerated conditions until analysis, which was performed in October 2021.

Sample digestion and subsequent trace element analyses of the maize flour samples were carried out at the Central Research Laboratory Application and Research Center of Recep Tayyip Erdogan University. Approximately 0.40 g of the powdered sample was transferred into Teflon digestion vessels. Subsequently, 5.0 mL of 65% HNO_3_ and 3.0 mL of 30% H_2_O_2_ were added to the digestion vessels for acid digestion [18]. The vessels were carefully shaken and allowed to stand for at least 20 min before being sealed. Thereafter, the vessels were tightly closed and placed in a microwave digestion system (Berghof Speedwave MWS-3+, Eningen, Germany). The digestion was carried out according to the temperature program described in Table 1. After the digestion process was completed, the vessels were left to cool to ambient temperature. The resulting solutions were subsequently filtered into 25 mL Falcon volumetric tubes and brought to volume with ultrapure water. The blank sample was prepared in parallel using the same digestion and dilution procedure.

**Table 1:** Temperature conditions employed in the microwave digestion procedure

Step	1	2
T (°C)	145	190
P (Bar)	50	50
Power (%)	70	90
Ta (min.)	10	5
Time (min.)	5	10

### Metal Analysis

Inductively Coupled Plasma–Optical Emission Spectrometry (ICP-OES; Optima 7000 DV, PerkinElmer, USA) was employed for the determination of Fe, Zn, Cu, Ni, Mn, Cd, and Cr concentrations in the maize flour samples [19]. Each maize flour sample was analyzed in triplicate to ensure the accuracy and reproducibility of the analytical results. The operating conditions of the ICP-OES instrument used for the determination of the analyzed elements are summarized in Table 2. Calibration standards were prepared from a 1000 mg/L ICP multi-element stock solution (Merck, Germany). Prior to analysis, instrumental tuning and optimization procedures were performed according to the manufacturer’s recommendations to ensure plasma stability and signal sensitivity. All measurements were carried out under optimized instrumental conditions. The selected analytical wavelengths for the analyzed elements are presented in Table 3.

**Table 2:** ICP-OES operating conditions used for trace element analysis

Parameter	Value/Description
RF power	1450 W
Plasma gas flow	17 L/min
Auxiliary gas flow	0.2 L/min
Nebulizer gas flow	0.8 L/min
Plasma viewing	Axial/radial
Peristaltic pump speed	1.5 mL/min
Nebulizer type	Concentric
Spray chamber	Cyclonic
Number of replicates	3
Purge	Normal
Resolution	Normal
Read delay	45 s
Wash time	60 s
Sample flow rate	1.5 mL/min
Wavelength range	160–900 nm
Detector	CCD array detector

**Table 3:** Analytical wavelengths, R^2^, LOD, and LOQ values of the analyzed elements

Element	Wavelength line (nm)	LOD (mg kg^‒1^)	LOQ (mg kg^‒1^)	*R* ^2^
Fe	238.204	0.01250	0.04125	0.9999
Zn	206.200	0.01250	0.04125	0.9998
Cu	327.393	0.05625	0.18562	0.9997
Ni	231.604	0.02500	0.08250	0.9995
Mn	257.610	0.00187	0.00620	0.9998
Cd	228.802	0.00437	0.01442	0.9993
Cr	267.716	0.01562	0.05155	0.9994

*LOD* limit of detection, *LOQ* limit of quantification, *R*^*2*^ Coefficient of Determination

### Method Validation and Quality Control

Method validation and quality control (QC) procedures were performed to ensure the accuracy, precision, and reliability of the ICP-OES measurements. Blank solutions, matrix blanks, calibration standards, and certified reference materials (CRM) were regularly analyzed throughout the analytical sequence. Continuing calibration verification (CCV) analyses and blank controls were performed after every ten samples to monitor instrumental stability, calibration performance, and potential contamination during the measurements [20]. In addition, QC monitoring solutions were periodically analyzed to evaluate possible calibration drift and instrumental deviations.

Quality control analyses were carried out using IAEA-336 (Lichen), a certified reference material supplied by the International Atomic Energy Agency (IAEA), Austria. The CRM was regularly analyzed during the analytical procedure and used to assess analytical accuracy, method performance, possible interferences, and calibration deterioration [20]. The calculated recoveries for the analyzed metals in the reference material ranged from 98.0% to 110.5%. The relative standard deviation (RSD) values obtained for all analyzed elements in the reference material were below 5%, indicating good analytical precision [21]. The certified values, measured concentrations, recovery percentages, and RSD values for the reference material are presented in Table 4.

**Table 4:** Results obtained for the certified reference material (IAEA-336)

Element	Recommended value (mg/kg)	95% Confidence interval (mg/kg)	Measured value (mg/kg)	Recovery (%)	RSD (%)
Fe	430	380–480	421.5	98.0	0.83
Zn	30.4	27.0–33.8	33.6	110.5	1.26
Cu	3.6	3.1–4.1	3.9	108.3	2.45
Mn	63	56–70	68.9	109.4	1.32
Cd	0.117	0.100–0.134	0.12	102.6	4.29
Cr	1.06	0.89–1.23	1.1	103.8	2.87

*RSD* relative standard deviation

The calibration curves for all analyzed trace elements exhibited good linearity, confirming the reliability of the analytical procedure. The high coefficient of determination (R^2^) values indicated a strong linear correlation between the measured responses and the analyte concentrations [22, 23]. The limits of detection (LOD) and limits of quantification (LOQ) were determined based on blank measurements. For this purpose, the blank solution was analyzed ten times, and the standard deviation of the blank signals (σ) was calculated. The LOD and LOQ values were calculated using the following equations: $\:\mathrm{L}\mathrm{O}\mathrm{D}=\left(3\times\:{\upsigma\:}\right)/\mathrm{S}$ and $\:\mathrm{L}\mathrm{O}\mathrm{Q}=\left(10\times\:{\upsigma\:}\right)/\mathrm{S}$ where σ represents the standard deviation of the blank measurements and S is the slope of the calibration curve. Accordingly, the LOQ values were approximately 3.3 times higher than the corresponding LOD values [24]. The LOD, LOQ, and R^2^ values for all analyzed elements are presented in Table 3.

### Non-Carcinogenic Health Risk Assessment

Non-carcinogenic health risks for adults were assessed through the estimation of average daily dose (ADD), hazard quotient (HQ), and hazard index (HI). The ADD (mg/kg/day) was calculated using the following equation to estimate human exposure resulting from the direct consumption of maize flour as bread or porridge [25, 26]:

1 $$\:\mathrm{A}\mathrm{D}\mathrm{D}=\frac{\mathrm{C}\times\:\mathrm{I}\mathrm{R}\times\:\mathrm{E}\mathrm{F}\times\:\mathrm{E}\mathrm{D}}{\mathrm{B}\mathrm{W}\times\:\mathrm{A}\mathrm{T}}$$

where C is the concentration of the corresponding trace element (mg/kg) and IR is the ingestion rate (kg/day). The per capita maize consumption in Turkey was reported to be 13.2 kg/year during the 2021–2022 period [27]. Accordingly, the daily maize consumption was estimated to be 0.036 kg/day. BW represents the average body weight of an adult, assumed to be 70 kg [28, 29]. EF refers to the exposure frequency, and ED refers to the exposure duration. AT corresponds to the averaging time for non-carcinogenic effects and is expressed as AT = EF × ED. In this study, the EF and ED values were assumed to be 365 days/year and 70 years, respectively, as commonly applied in human health risk assessments [28, 30].

To assess non-carcinogenic health risks posed by trace elements, the hazard quotient (HQ) and hazard index (HI) were calculated using the following equations, with HI calculated as the sum of the HQ values of all examined metals [25, 26]:

2 $$\:\mathrm{H}\mathrm{Q}=\frac{\mathrm{A}\mathrm{D}\mathrm{D}}{\mathrm{R}\mathrm{f}\mathrm{D}}$$

3 $$\:\mathrm{H}\mathrm{I}=\sum\:_{\mathrm{i}=1}^{5}{\mathrm{H}\mathrm{Q}}_{\mathrm{i}}$$

where RfD represents the daily oral reference dose. The RfD values for the examined elements (Fe, Zn, Cu, Ni, and Mn) were reported as 0.7, 0.3, 0.04, 0.02, and 0.14 mg/kg/day, respectively, according to the United States Environmental Protection Agency (USEPA) guidelines [31].

### Statistical Analysis

Statistical analyses were performed using SPSS (version 23.0) and OriginPro (version 9.0) for Windows. Descriptive statistical analyses were used to summarize the concentrations of Fe, Zn, Cu, Ni, and Mn in the maize flour samples. Spearman’s rank correlation analysis was conducted to evaluate the relationships among the analyzed elements, and statistical significance was set at *p* < 0.05. In addition, Principal Component Analysis (PCA) was performed to investigate the distribution patterns and possible grouping of the trace elements.

## Results and Discussion

### Trace Element Concentrations in Maize Flour Samples

**Fig. 1:**
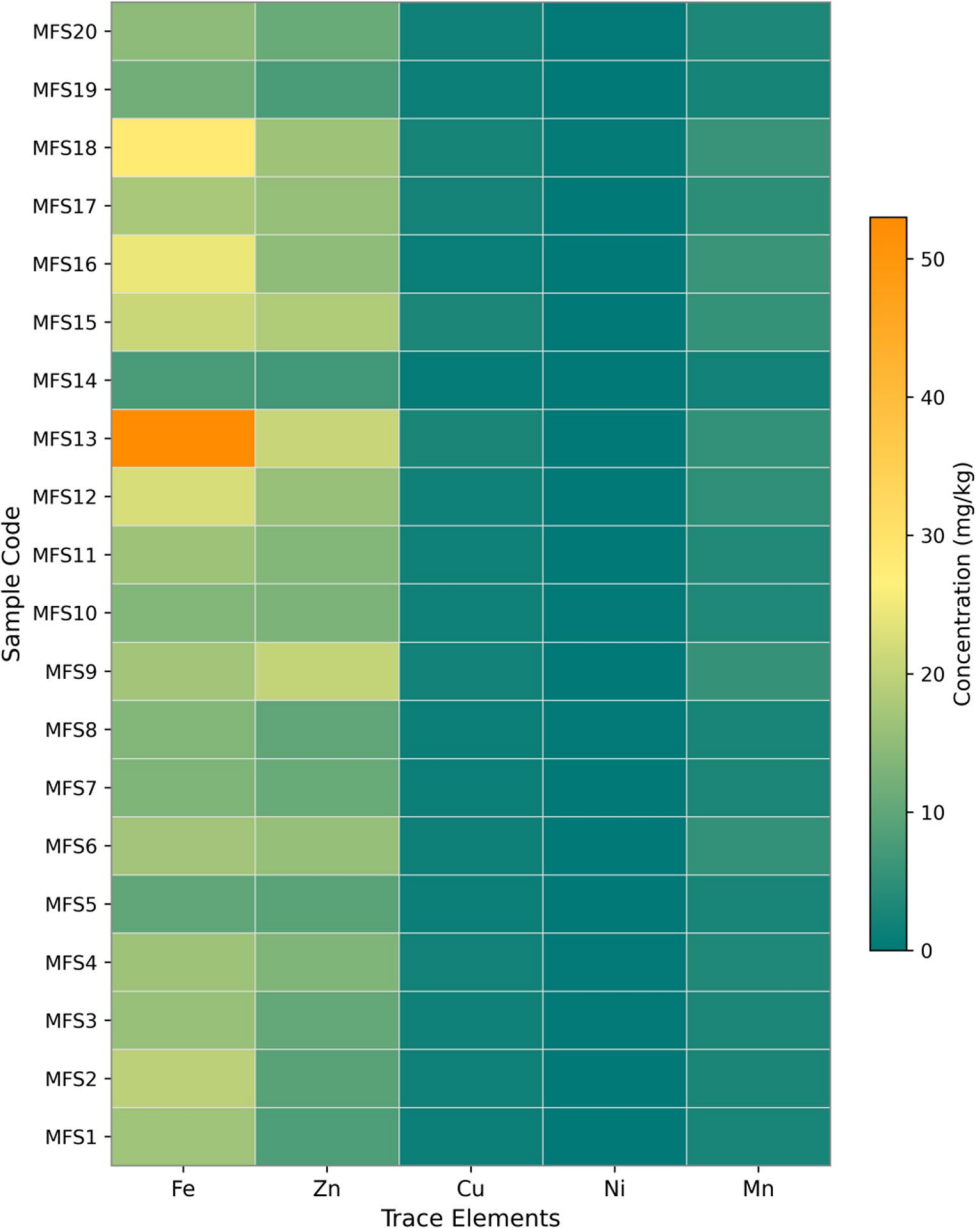
Heatmap showing the distribution of analyzed trace element concentrations in the maize flour samples

The distribution of Fe, Zn, Cu, Ni, and Mn concentrations in the maize flour samples is illustrated in Fig. 1. Fe is an essential element involved in oxygen transport and various metabolic processes; however, excessive intake may lead to oxidative stress and tissue damage [32]. In the present study, Fe concentrations ranged from 7.7 to 53.0 mg/kg, with a mean value of 18.5 mg/kg. The mean Fe concentration exceeded the permissible limit of 15 mg/kg [33]. Mn is an essential element involved in several biological and enzymatic processes; however, excessive intake may result in toxic effects, particularly on the nervous system [34]. Mn concentrations ranged from 1.9 to 6.2 mg/kg, with a mean value of 3.9 mg/kg. Except for MFS14, all samples exceeded the permissible limit of 2.3 mg/kg [35], while MFS19 was at the permissible limit.

Fe and Mn are essential elements required for plant growth and are primarily taken up by plant roots from the rhizosphere. Since both elements naturally occur in soil minerals, their concentrations in maize flour samples may partly be associated with lithogenic sources and soil geochemistry [36–39]. Additionally, atmospheric deposition and subsequent foliar uptake may contribute to the accumulation of Fe and Mn in maize plants during cultivation, potentially influencing the elemental concentrations observed in the flour samples [40–42]. Irrigation practices may also influence Fe and Mn levels depending on the geochemical composition of irrigation water [43, 44]. Furthermore, processing-related contamination should also be considered, as metallic wear from milling equipment during flour production may contribute to elevated Fe and, to a lesser extent, Mn concentrations in flour products [26, 45, 46].

Zn is an essential trace element involved in numerous enzymatic, metabolic, and immune functions; however, excessive intake may interfere with copper metabolism and immune function [47]. In the present study, Zn concentrations ranged from 6.9 to 20.8 mg/kg, with a mean value of 13.1 mg/kg. All measured Zn concentrations remained below the permissible limit of 30 mg/kg [33]. Cu is an essential element required for many biological functions; however, excessive intake may lead to liver and neurological disorders [48]. Cu concentrations ranged from 0.8 to 2.9 mg/kg, with a mean value of 1.7 mg/kg, and all measured concentrations remained below the permissible limit of 40 mg/kg [26]. Ni is a widely distributed environmental trace metal that may cause allergic and neurological effects at elevated exposure levels [49]. In this study, Ni concentrations were generally low. Ni was not detected in five samples, while the highest concentration was 0.6 mg/kg, with a mean value of 0.2 mg/kg. All measured Ni concentrations remained below the permissible limit of 10 mg/kg [35].

### Comparison of Trace Element Concentrations with Previous Studies

Table 5 presents a comparison of trace element concentrations reported in maize flour samples from different countries using various analytical techniques. Fe concentrations differed considerably among studies. The mean Fe concentration determined in the present study (18.5 mg/kg) was close to the values reported for Uganda [26] and Ghana [50], but lower than those reported for Egypt [2], Kenya [17], and Pakistan [53]. In contrast, it was higher than the values reported for Brazil [1] and Cape Verde [54]. Zn concentrations also exhibited substantial variation among studies. The mean Zn concentration obtained in the present study (13.1 mg/kg) was close to the values reported for Egypt [2], Uganda [26], and Turkey [52]. However, it was lower than the values reported for Kenya [17] and Pakistan [53], while being higher than several previously reported values.

**Table 5:** Comparison of mean trace element concentrations (mg/kg) in maize flour samples with those reported in the literature

Country	Method	Fe	Zn	Cu	Ni	Mn	References
Brazil	FAAS	7.82	2.18	0.52	–	1.35	[1]
Ghana	FAAS	20.44	6.04	0.03	**–**	**–**	[50]
Ghana	AAS	1.3392	0.6809	**–**	0.9502	0.3550	[25]
Uganda	AAS	18.89	11.27	1.37	0.69	11.68	[26]
Kenya	TXRF	44.5	30.7	2.8	–	3.7	[17]
Egypt	Q-ICP-MS	42.49	12.39	1.54	0.53	5.07	[2]
Brazil	ICP-MS	**–**	9.0	1.0	42	3.1	[51]
Turkey	ICP-MS	**–**	15.37	1.38	**–**	**–**	[52]
Pakistan	ICP-MS	115.13	33.898	11.465	6.083	21.481	[53]
Cape Verde	ICP-OES	11.5	5.58	0.70	**–**	2.55	[54]
Turkey	ICP-OES	18.5	13.1	1.7	0.2	3.9	This Study

The Cu concentration determined in this study (1.7 mg/kg) was close to the values reported for Egypt [2], Uganda [26], Brazil [51], and Turkey [52]. It was higher than the values reported for Brazil [1], Ghana [50], and Cape Verde [54], but lower than that reported for Kenya [17] and Pakistan [53]. Ni concentrations were reported in fewer studies, and the mean Ni concentration in the present study (0.2 mg/kg) was lower than the values reported in the available literature. Mn concentrations also varied among studies. The mean Mn concentration in this study (3.9 mg/kg) was close to the values reported for Kenya [17], Brazil [51], and Cape Verde [54], but lower than those reported for Egypt [2], Uganda [26], and Pakistan [53]. In contrast, it was higher than the values reported for Brazil [1] and Ghana [25].

Overall, the differences observed among studies indicate that trace element concentrations in maize flour may be influenced by several factors, including environmental conditions, agricultural practices, milling processes, sample origin, and analytical techniques used for trace element determination [54–57].

### Assessment of Non-carcinogenic Health Risks

**Fig. 2:**
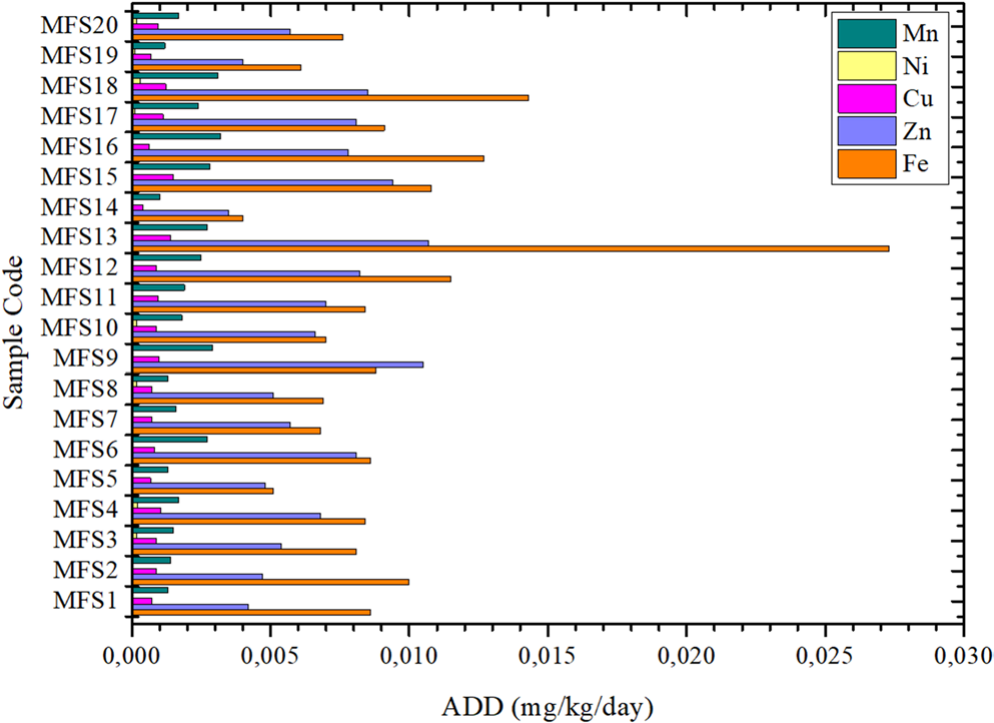
Element-specific average daily dose (ADD) values in the maize flour samples

The estimation of average daily dose (ADD), hazard quotient (HQ), and hazard index (HI) values were calculated to assess the non-carcinogenic health risks associated with the trace element concentrations in the analyzed maize flour samples. The ADD values obtained for the investigated trace elements are presented in Fig. 2. The minimum, maximum, and mean ADD values for Fe were determined as 4.0 × 10^–3^, 27.3 × 10^–3^, and 9.5 × 10^–3^ mg/kg/day, respectively. For Zn, the corresponding values were 3.5 × 10^–3^, 10.7 × 10^–3^, and 6.7 × 10^–3^ mg/kg/day, respectively. The minimum, maximum, and mean ADD values of Cu were found to be 4.1 × 10^–4^, 14.9 × 10^–4^, and 9.0 × 10^–4^ mg/kg/day, respectively. In the case of Ni, the maximum and mean ADD values were determined as 30.9 × 10^–5^ and 8.5 × 10^–5^ mg/kg/day, respectively. Similarly, Mn exhibited minimum, maximum, and mean ADD values of 1.0 × 10^–3^, 3.2 × 10^–3^, and 2.0 × 10^–3^ mg/kg/day, respectively. In the present study, the mean ADD values for all examined elements were found to be below their respective RfD values as defined by the USEPA guidelines [31].

The estimated daily intake values per person for Fe, Zn, Cu, Ni, and Mn in the present study, calculated based on the mean ADD values, were determined to be 6.7 × 10^–1^, 4.7 × 10^–1^, 6.3 × 10^–2^, 5.9 × 10^–3^, and 1.4 × 10^–1^ mg/day, respectively. These mean daily intake values were lower than the corresponding values of 2.1800, 0.6357, 0.0788, 0.0274, and 0.2601 mg/day reported by Al-Trbany et al. for Fe, Zn, Cu, Ni, and Mn, respectively [2]. Similarly, the mean daily intake values of Fe, Zn, Cu, and Mn obtained in the present study were lower than the corresponding values of 1.15, 0.56, 0.07, and 0.26 mg/day reported by Rubio-Armendáriz et al. for Fe, Zn, Cu, and Mn, respectively [54]. Furthermore, the values determined in this study were considerably lower than the minimum daily intake values of 4.5, 3.7, 0.4, and 0.5 mg/day reported by Maina et al. for Fe, Zn, Cu, and Mn, respectively [17].

**Fig. 3:**
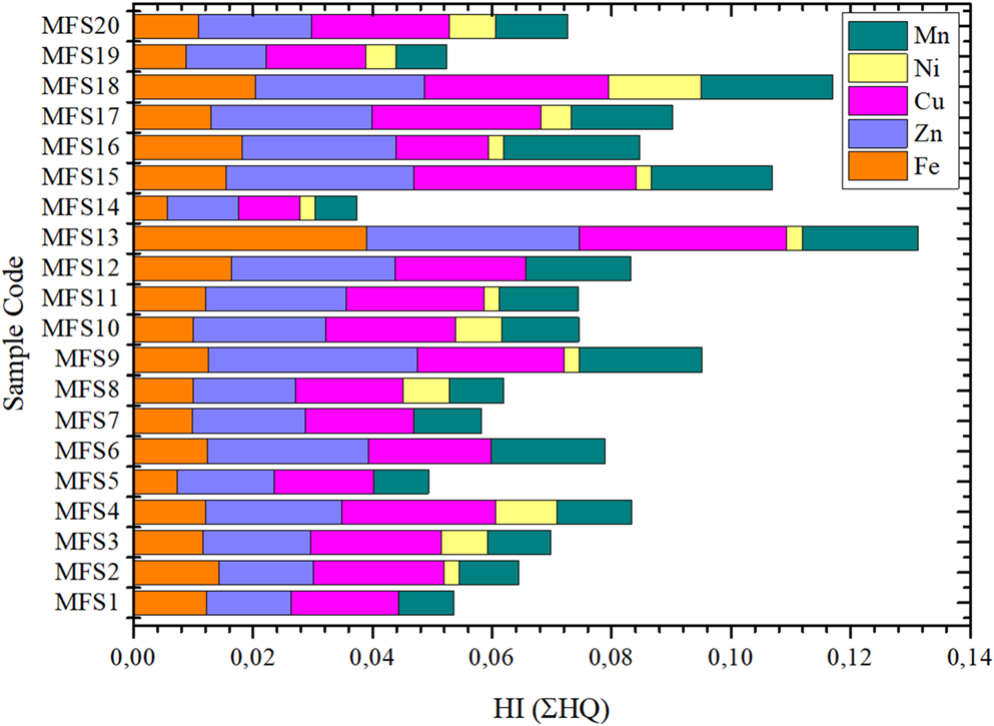
Contributions of element-specific hazard quotient (HQ) values to the hazard index (HI) in the maize flour samples

The HQ values of the investigated trace elements and the HI values calculated from HQ contributions for the maize flour samples are presented in Fig. 3. The HQ values varied as follows: Fe, from 0.6 × 10^–2^ to 3.9 × 10^–2^, with a mean of 1.4 × 10^–2^; Zn, from 1.2 × 10^–2^ to 3.6 × 10^–2^, with a mean of 2.3 × 10^–2^; Cu, from 1.0 × 10^–2^ to 3.7 × 10^–2^, with a mean of 2.2 × 10^–2^; Ni, with a maximum of 1.5 × 10^–2^ and a mean of 0.4 × 10^–2^; and Mn, from 0.7 × 10^–2^ to 2.3 × 10^–2^, with a mean of 1.4 × 10^–2^. The HI values ranged from 3.7 × 10^–2^ in sample MF-14 to 13.1 × 10^–2^ in sample MF-13, with a mean of 7.7 × 10^–2^. These findings indicate that although these elements are ingested through the consumption of the maize flour analyzed in this study, all HQ and HI values remained well below 1, demonstrating that none of the samples represent a non-carcinogenic health risk.

### Statistical Analysis

#### Descriptive Statistics

**Fig. 4:**
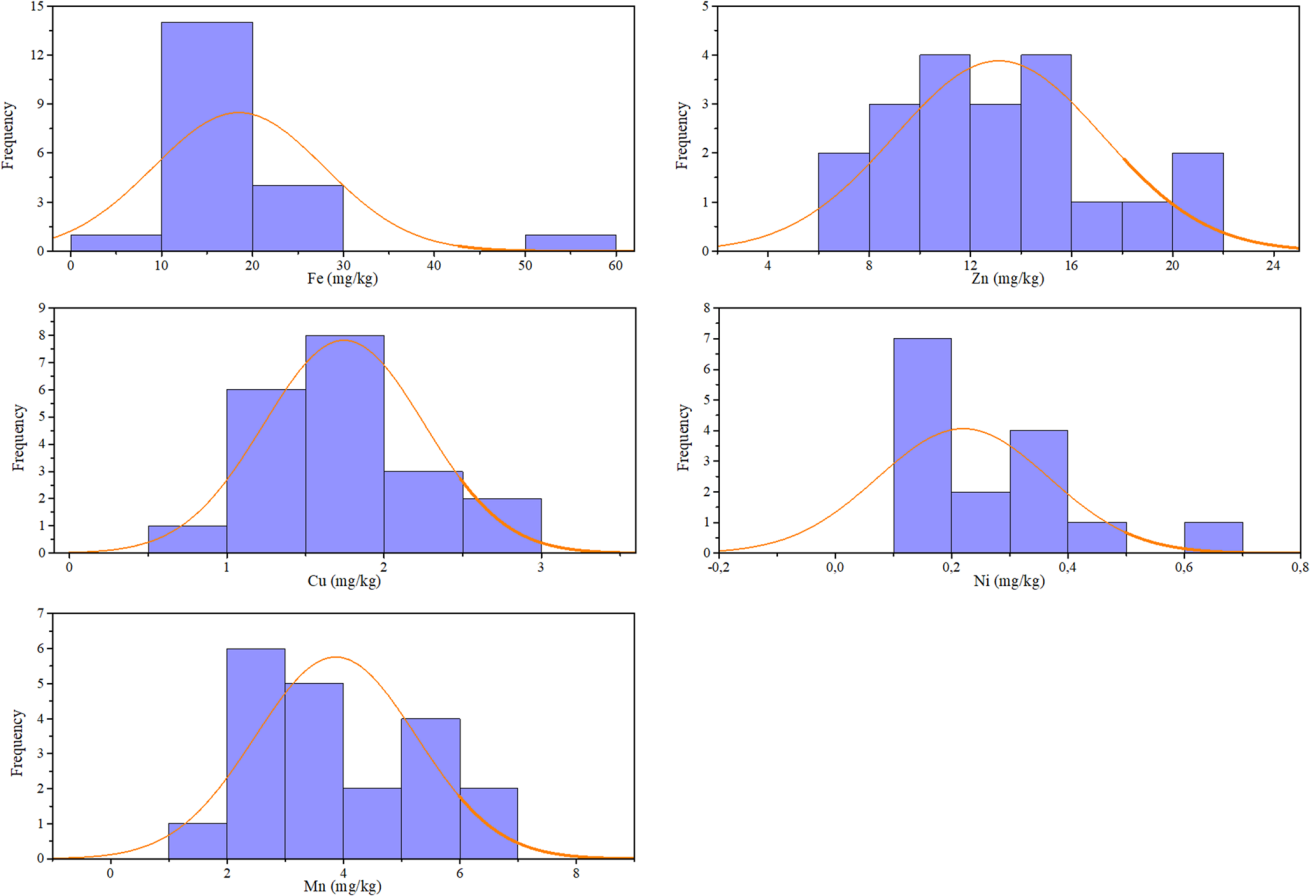
Frequency distributions of Fe, Zn, Cu, Ni, and Mn concentrations in the maize flour samples

Descriptive statistical methods were used to summarize the distributional characteristics of trace element concentrations in the analyzed maize flour samples. Table 6 presents a descriptive statistical overview of trace element concentrations in the maize flour samples, including parameters such as the mean, median, variance, standard deviation, minimum and maximum values, range, skewness, and kurtosis. The normality of data distributions was evaluated using the Shapiro–Wilk test, with additional support from skewness and kurtosis coefficients. A significance threshold of 0.05 was adopted for the Shapiro–Wilk analysis. Based on the results, Zn, Cu, and Mn concentrations exhibited normal distribution patterns (*p* > 0.05), whereas Fe and Ni concentrations deviated from normality (*p* < 0.05). In general, distributions are considered approximately normal when skewness and kurtosis values approach zero [58, 59]. Based on this criterion, the skewness and kurtosis values calculated for Fe, Zn, Cu, Ni, and Mn indicate that Zn, Cu, and Mn exhibit a normal distribution, whereas Fe and Ni do not follow a normal distribution. Additionally, the frequency distributions of Fe, Zn, Cu, Ni, and Mn concentrations are shown in Fig. 4. As observed, Zn, Cu, and Mn concentrations exhibit an approximately normal distribution, while Fe and Ni concentrations depart from normality. The differences observed in Fe and Ni levels in maize flour samples may be associated with variability in the amounts of metals transferred into the flour as a result of mill plate wear during the milling process, initial metal accumulation in the grains during plant growth, and the operational condition of the milling equipment. The influence of these factors may have resulted in heterogeneous distributions of Fe and Ni concentrations among the samples and, consequently, deviations from a statistically normal distribution [26].

**Table 6:** Descriptive statistical overview of trace element concentrations in the maize flour samples

Sample code	Fe	Zn	Cu	Ni	Mn
Mean	18.5	13.1	1.7	0.2	3.9
Median	16.6	13.1	1.7	0.2	3.5
Variance	88.6	16.9	0.3	0.0	1.9
Std. Deviation	9.4	4.1	0.5	0.1	1.4
Minimum	7.7	6.9	0.8	–	1.9
Maximum	53.0	20.8	2.9	0.6	6.2
Range	45.3	13.9	2.1	0.5	4.3
Skewness	2.8	0.3	0.7	1.3	0.3
Kurtosis	9.8	–0.8	0.6	1.7	–1.4

#### Spearman’s Rank Correlation Analysis

Table 7 presents the Spearman’s rank correlation coefficients (ρ) between the trace elements Fe, Zn, Cu, Ni, and Mn in maize flour samples. The analysis revealed strong positive correlations among Fe, Zn, and Mn, with ρ values of 0.756 (Fe–Zn), 0.809 (Fe–Mn), and 0.931 (Zn–Mn), suggesting a potential common source or similar accumulation behavior. Cu exhibited moderate correlations with Fe (ρ = 0.603), Zn (ρ = 0.730), and Mn (ρ = 0.595), indicating partial association with these elements. In contrast, Ni showed weak correlations with the other elements (ρ ranging from 0.265 to 0.500), implying a distinct origin or independent distribution pattern. Overall, these findings highlight that Fe, Zn, and Mn are closely associated, whereas Ni likely originates from a separate source in the maize flour samples. Fe, Zn, Mn, and Cu are widely recognized as essential micronutrients for plant growth and metabolism, serving as catalytic cofactors and enzyme activators in physiological processes such as photosynthesis and nutrient metabolism. These elements are required in small amounts but are crucial for normal plant development and biochemical function [60, 61]. In contrast, Ni, although listed as a micronutrient in some sources, is required at very low concentrations and can become toxic at elevated levels, causing adverse effects on plant physiological processes [62]. This supports the observation that Ni’s accumulation pattern may differ from that of the essential elements in maize flour samples.

**Table 7:** Spearman’s rank correlation coefficients of the elements

	Fe	Zn	Cu	Ni	Mn
Fe	1				
Zn	0.756^**^	1			
Cu	0.603^**^	0.730^**^	1		
Ni	–0.024	0.006	0.368	1	
Mn	0.809^**^	0.931^**^	0.595^**^	0.265	1

^**^Correlation is significant at the 0.01 level (2-tailed)

#### Principal Component Analysis (PCA)

**Fig. 5:**
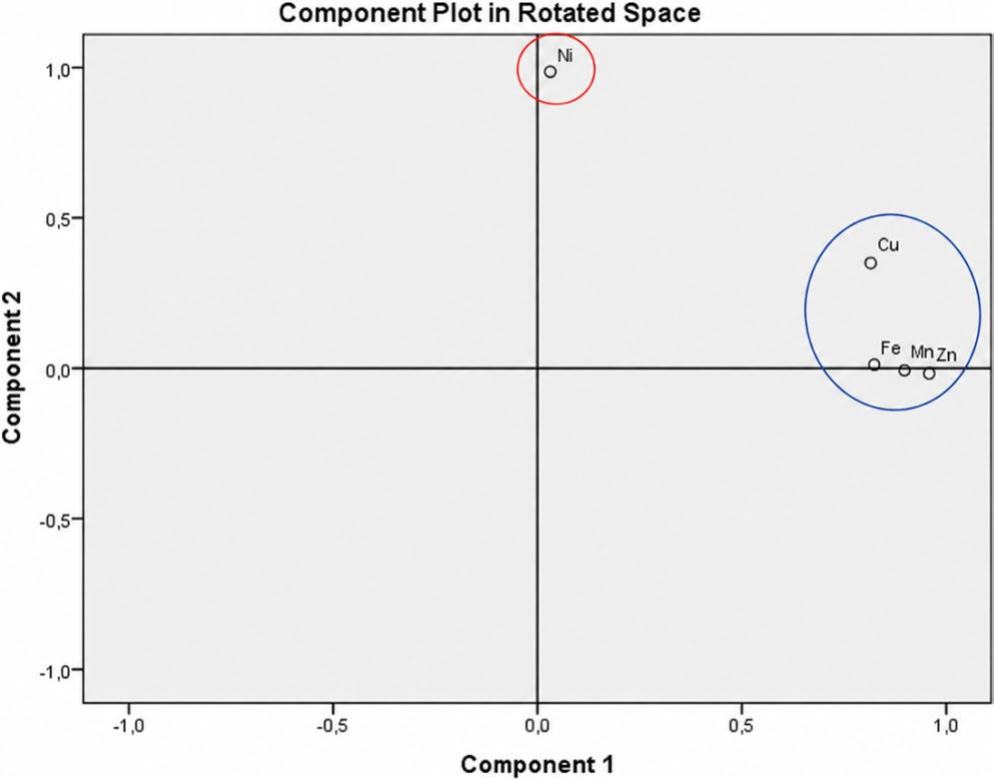
PCA component loadings of trace elements in rotated space

Principal Component Analysis (PCA) was performed to evaluate the relationships among trace elements, identify contamination patterns, and determine potential pollution sources. The rotated component loadings obtained from PCA are presented in Table 8, while the component loading plot in rotated space is shown in Fig. 5. According to the PCA results, two principal components with eigenvalues greater than 1 were extracted. PC1 was mainly associated with Fe, Zn, Cu, and Mn, with high positive loading values of 0.824, 0.959, 0.816, and 0.898, respectively. The close association among these elements suggests that they may originate from similar sources or exhibit comparable accumulation behaviors. In contrast, Ni exhibited a strong positive loading on PC2 (0.986) and was clearly separated from the other trace elements in the rotated component plot (Fig. 5). This separation indicates that Ni may originate from a different source or exhibit distinct distribution characteristics compared to the other elements. Furthermore, the grouping of Fe, Zn, Cu, and Mn within the same region of the loading plot indicates strong associations among these elements. These associations may be attributed to similar environmental conditions, agricultural practices, processing-related factors, and geographic proximity, which may affect the elemental composition of the maize flour samples [1, 57, 63, 64].

**Table 8:** Rotated component matrix of trace elements

Variables	PC1	PC2
Fe	0.824	0.011
Zn	0.959	–0.016
Cu	0.816	0.349
Ni	0.032	0.986
Mn	0.898	–0.007

## Conclusion

The concentrations of trace elements (Fe, Zn, Cu, Ni, Mn, Cd, and Cr) in maize flour samples commonly consumed in Turkey were determined using inductively coupled plasma optical emission spectrometry (ICP-OES). The measured concentrations were compared with international guideline values and previously reported literature data. Descriptive statistical analysis was used to summarize the concentration levels of the analyzed elements. Spearman’s rank correlation analysis was performed to evaluate the relationships among trace elements, while Principal Component Analysis (PCA) was used to identify possible contamination patterns and potential pollution sources. The PCA results showed that Fe, Zn, Cu, and Mn were grouped within the same principal component, indicating strong associations among these elements, whereas Ni exhibited distinct behavior compared to the other elements. The findings revealed that the mean concentrations of Fe and Mn exceeded their respective permissible limits, and a statistically significant correlation was observed between these two elements. In contrast, Cd and Cr concentrations in all analyzed samples were below the detection limits. Potential non-carcinogenic health risks associated with dietary exposure to these elements were also evaluated using the calculated average daily dose (ADD), hazard quotient (HQ), and hazard index (HI) values based on the estimated daily intake for adult consumers. The HQ and HI values for all analyzed elements were below 1, indicating that maize flour consumption is unlikely to pose significant non-carcinogenic health risks to adults at the observed concentration levels. Nevertheless, the findings are limited to the maize flour samples analyzed in the present study. Furthermore, the analytical approach employed in this study may also be applicable to other cereal-based products and different food matrices. The proposed methodology may contribute to future trace element monitoring studies in different geographical regions and provide useful data for dietary exposure and food safety assessments.

## Data Availability

No datasets were generated or analysed during the current study.
